# Autosomal dominant monilethrix with incomplete penetrance due to a novel *KRT86* mutation in a Chinese family^☆^

**DOI:** 10.1016/j.abd.2022.12.010

**Published:** 2024-04-08

**Authors:** Ru Dai, Tingting Wang, Xianjie Wu

**Affiliations:** Department of Dermatology, Zhejiang University School of Medicine Second Affiliated Hospital, Hangzhou, Zhejiang, China

Dear Editor,

Monilethrix (OMIM 158000), also known as beaded hair, is a rare hereditary hair disorder, characterized by abnormal hair shafts with periodic nodes and internodes, hair fragility, follicular hyperkeratosis, and sparseness of hair.^1^ Classically, it is caused by autosomal dominant mutations in basic hair keratin genes *KRT86*, *KRT83* and *KRT81*.^2^ Rarely, an autosomal recessive mutation in the *DSG4* gene may contribute to the disease.^3^ Here, we present a two-generation Chinese family with autosomal dominant monilethrix due to a novel heterozygous missense mutation in *KRT86* (c.1226T>C, p.Leu409Pro).

The proband (II-2) was a 30-year-old woman. She developed sparse, short, and fragile hairs with apopecia since infancy (Fig. 1A). There were numerous keratotic follicular papules on her occipital area (Fig. 1B). The secondary hair, eyebrow, eyelashes, fingernails, and systemic examination were all normal. Dermoscopic examination showed typical beading and nodes (Fig. 1C). Under light microscopy, the hair shaft showed characteristic elliptical nodes and intermittent constrictions (Fig. 2A). Scanning electron microscopy revealed that cylindrical hair had a segmental structure with periodic nodules and narrow parts: width of the nodules was 0.09‒0.11 mm and width of the constriction was 0.05‒0.08 mm. The parallel longitudinal ridge and groove could be seen on the surface similar to the bark-like appearance, and an erosion-like structure appeared on the cross-section (Fig. 2B). Histopathological examination of the affected scalp showed hyperkeratosis, decreased hair follicle density, infiltration of chronic inflammatory cells around the follicular unit with plugging (Fig. 3).

**Figure 1:**
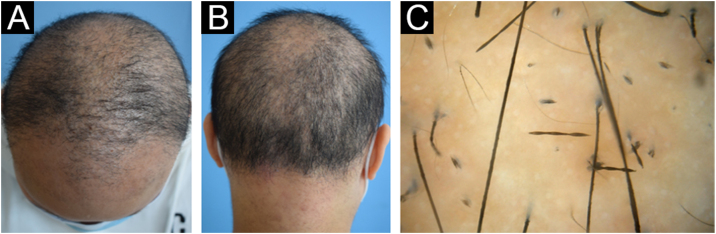
Clinical features and dermoscopy of the proband. The proband exhibited sparse hair (A) and follicular hyperkeratosis (B). Dermoscopy of the proband showed typical beading and nodes (C).

**Figure 2:**
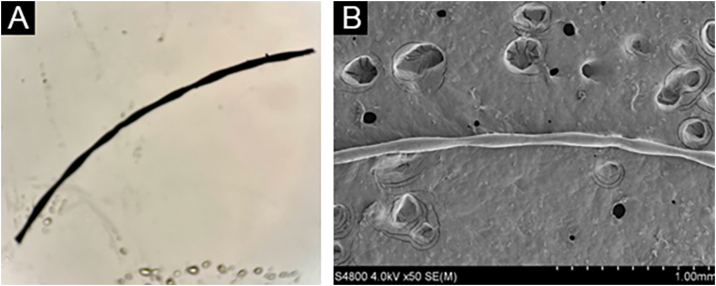
Microscopy and scanning electron microscopy of the proband. (A) Light microscopic showed characteristic elliptical nodes and intermittent constriction. (B) Scanning electron microscopy revealed that cylindrical hair had a segmental structure with periodic nodules and narrow parts.

**Figure 3:**
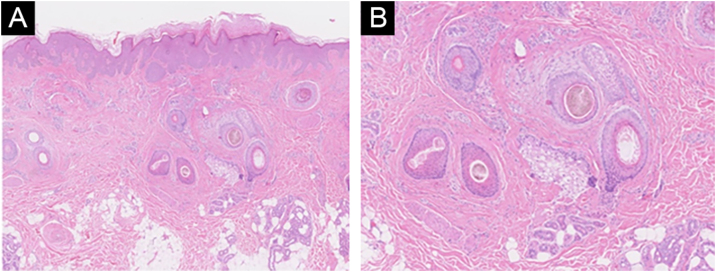
Histologic feature of the proband. Histopathology examination of the affected scalp showed hyperkeratosis, decreased hair follicle density, infiltration of chronic inflammatory cells around the follicular orifice with plugging (Hematoxylin & eosin, [A]×50, [B]×100).

Her father aged 58 years also had noticeable hair loss with less marked follicular papules (Fig. 4A‒B). Dermoscopy revealed hair fragility and breakage (Fig. 4C). Her younger brother aged 17 years was born with full hair and seemed to have a normal hair appearance, while his hairs were also coarse and lusterless with slight follicular hyperkeratosis on the scalp. Dermoscopy revealed apparent moniliform hair. Her mother had normal hair on clinical and dermoscopic examination.

**Figure 4:**
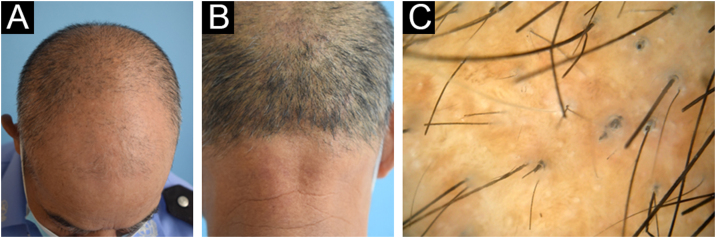
Clinical features and dermoscopy of the fathers' patient. The father exhibited sparse hair (A) without follicular hyperkeratosis (B). Dermoscopy of the father revealed hair fragility and breakage (C).

After obtaining written informed consent, peripheral blood samples were taken from the family for Whole-Exome Sequencing (WES). The WES result showed a novel heterozygous missense mutation (c.1226T>C, p.Leu409Pro) in exon 7 of the *KRT86* gene in all three affected family members (Fig. 5), which resulted in a leucine to proline substitution.

**Figure 5:**
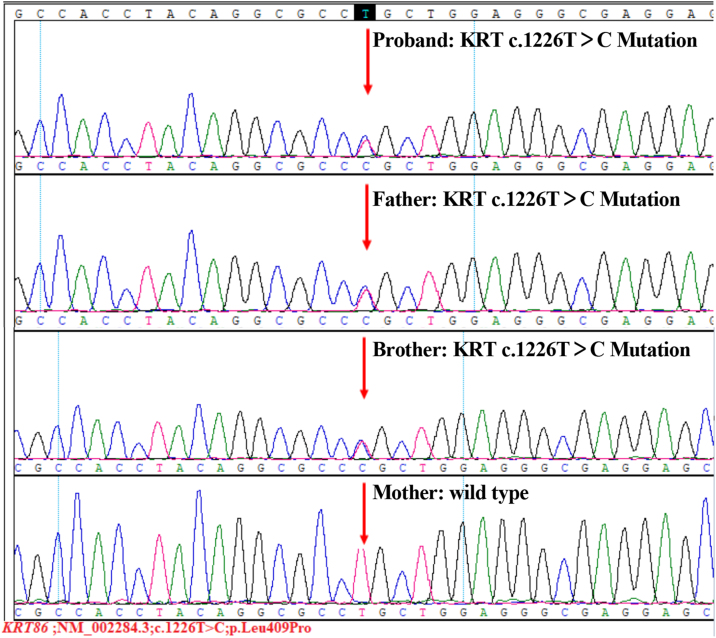
The sequence of the heterozygous mutation in *KRT86* gene. The proband, her father and her brother all had a heterozygous T to C mutation (c.1226T＞C, p.Leu409Pro) in the exon 7 of *KRT86*. The sequence of mother was normal.

Monilethrix is a structural defect of the hair shaft, usually caused by mutations in genes encoding hair keratins. *KRT86* and *KRT81* are the most common involved genes.^4^ In the present study, the identified mutation c.1226T>C in *KRT86* leads to the substitution of leucine to proline, thereby affecting the keratin intermediate filament assembly and stability. The variant has not been reported previously in the literature database or in the ClinVar database. To our knowledge, this is also the first time that this mutation has been demonstrated causing monilethrix, which extends the spectrum of *KRT86* mutations. However, the precise mechanisms for the moniliform hair remain to be elucidated. Incomplete penetrance was a striking feature of this family. Among affected family members severity of the phenotype may vary from extreme alopecia to normal hair appearance.^5^ In our study, we presented a monilethrix family in which two members presented hair loss, and one was clinically unremarkable. The dermoscopy confirmed moniliform hairs in this family member. These findings support the clinical variability in monilethrix.

In summary, we presented here a new mutation c.1226T>C in exon 7 of *KRT86* in a two-generation Chinese family with monilethrix.

## Financial support

This research was funded by the Natural Science Foundation of China (nº 82103754).

## Authors’ contributions

Ru Dai: Made substantial contributions to the design of the manuscript, acquisition, analysis and interpretation of data; Draft and submit the manuscript; Read and approved the final manuscript.

Tingting Wang: Had been involved in the design and revision of the manuscript; Acquisition, analysis and interpretation of data; Read and approved the final manuscript.

Xianjie Wu: Reviewed the histologic, dermoscopic and scanning electron microscopic images; Reviewed the final manuscript and gave the final approved of the version to be submitted.

## Conflicts of interest

None declared.
